# Three types of phenomenal consciousness and their functional roles: unfolding the ALARM theory of consciousness

**DOI:** 10.1098/rstb.2024.0314

**Published:** 2025-11-13

**Authors:** Albert Newen, Carlos Montemayor

**Affiliations:** ^1^ Institut für Philosophie II, Ruhr-Universität Bochum, 44780 Bochum, North Rhine-Westphalia, Germany; ^2^ Department of Philosophy, San Francisco State University, San Francisco, CA 94132, USA

**Keywords:** consciousness, function of consciousness, basic arousal, general alertness, reflexive consciousness

## Abstract

The evolution of consciousness is a neglected topic that plays a surprisingly insignificant role in all major theories of consciousness. Furthermore, substantial disagreements can be observed in the dominant views on the neural correlates of consciousness (NCCs), which focus too much on cortical brain regions. In order to dissolve some of the contradictions among these views and to constrain the rival theories, we propose to distinguish three core phenomena of phenomenal consciousness: basic arousal, general alertness and reflexive (self-)consciousness. The central aim is to show that we can fruitfully distinguish specific functions for each of the three phenomena. Basic arousal has the function to alarm the body and secure survival by intervening in the slow updating of homeostatic processes. General alertness fosters advanced learning and decision-making processes, enabling various new behavioural strategies to deal with challenges, and reflexive (self-)consciousness enables future-directed long-term planning, accounting for the mindset of oneself and other agents. Constraining our contemporary theories of consciousness with this evolutionary and functional approach will enable the science of consciousness to make progress by accounting for three specific functions of consciousness, thereby informing the search for distinct an NCC.

This article is part of the theme issue ‘Evolutionary functions of consciousness’.

## 1. Introduction

Evolution is typically discussed as a conundrum in the context of consciousness research, with authors defending a wide variety of views, with respect to both the demarcation problem of what species are conscious and the time during our evolution when consciousness first emerged [1–3]. Consciousness certainly is, as Lane [4] puts it, a great invention of evolution. Our lives would not be the same without it; it might even be inconceivable to imagine our lives without being conscious. Yet, because of the standard interpretation of the ‘hard problem’, most functional accounts, including evolutionary ones, are still considered as preliminary, at best. This situation of lack of empirical constraints concerning evolution is reflected in the core commitments of the major scientific views on consciousness, none of which takes evolution as a major factor in its explanation of consciousness. Moreover, the empirical predictions of these theories at the neural level are incompatible, making the situation worse. If, besides the scientific obfuscation produced by the hard problem, the leading scientific theories contradict each other, then it is not surprising that our scientific understanding of consciousness has not progressed much.

We believe that the recent and considerable progress in neuroimaging techniques needs to be accompanied by equally substantial theoretical progress regarding our understanding of the functionary roles that consciousness performed in the evolution of the minds. Evolution needs to become a major factor in our explanation of consciousness. We start by setting the stage with two observations that we take to be criteria of adequacy: first, we argue that non-cortical areas must be part of the explanation of consciousness, and that we can best characterize the non-cortical processing of consciousness as basic arousal (§1). Second, we need to distinguish three phenomena of consciousness, namely basic arousal, general alertness and reflexive self-consciousness (§2): those are different varieties of phenomenal consciousness. Since it is not clear whether reflexive self-consciousness should be included in the discussion of an evolutionary perspective on functions of consciousness (in addition to basic arousal and general alertness), we clarify the status of this phenomenon. We show that there are non-linguistic tests indicating the realization of reflexive self-consciousness in non-human animals (§3a). Furthermore, we argue that reflexive self-consciousness is best described as a special case of general alertness (§3b), but it nevertheless has a specific functional role going beyond the function of standard general alertness. With this background, we then unfold the main argument of the paper as follows: we show that an evolutionary perspective in combination with neuroscientific evidence supports (i) that three selected scientific theories of consciousness (information integration theory (IIT), higher-order-thought (HOT) and global neuronal workspace theory (GNWT)) either do not adequately account for the basic form of consciousness called basic arousal or do not consider it at all (§4), and (ii) that the three distinct phenomena of consciousness (basic arousal, general alertness and reflexive self-consciousness) have three different core functions (§5).

Let us set the stage with some methodological remarks: first, we do not discuss vegetative states or other preconscious states, but focus on phenomenal consciousness, in the sense of having a conscious experience in a state of wakefulness [5]. We also abstract in this context from the distinction between access and phenomenal consciousness [6] since we focus on phenomenal consciousness that is experienced longer than the duration of ultra-short-term memory, which is the focus of Block [6]. Second, we do not characterize types of consciousness as certain types of contents, e.g. higher-order theories are inclined to do this. We presuppose an independence of phenomenality and content in the sense that a phenomenal experience cannot be explained by a certain content, but only by the way a content is processed [7,8]. Third, we want to distinguish three ways of answering the question about the functional role of phenomenal consciousness [9]: (i) consciousness is the result of an adaptation; or (ii) it is an inseparable element of something else that is an adaptation; or (iii) it is a useless by-product, which means that it is an epiphenomenon. We are not discussing here epiphenomenalism, which is, e.g., a constitutive component of Chalmers’ dualism [10], because we think that there is convincing criticism for not going in the direction of epiphenomenalism (neither version of it) [11,12]. Furthermore, to discuss the function of consciousness, we will focus on the best candidates for a theoretical framework to investigate and understand consciousness, namely GNWT, IIT and HOT. We aim to show that they are missing a basic evolutionary perspective, namely what the earliest evolutionary functional role of consciousness could be. We want to close this gap but remain neutral about the debate about which of the three theories (if any) will win the open race about the best theoretical framework of consciousness. However, we claim that our evolutionary perspective is an important foundation for any successful and more detailed theory of consciousness in the future. Our fourth methodological commitment highlights the limits of our approach: we explicitly account for the co-evolutionary unfolding of consciousness in species with quite different brain structures, like birds and fish. Thus, we are not expecting one and the same cluster of neural mechanisms to be the realizer in all conscious living beings. With the ALARM theory of consciousness, we propose to start with the characterization of the most basic form of consciousness by describing its embeddedness in core principles of life, focusing on improving homeostatic regulation systems that keep the body alive (see below). Therefore, we propose a combination of a functional and neural characterization of the evolution of consciousness, and the result is our proposal for distinguishing three forms of phenomenal consciousness.

## 2. Two framework observations

### (a) Basic arousal is a fundamental type of consciousness realized by non-cortical processes

We propose that there is a level of consciousness that we call ‘basic arousal’ [8], which is biologically fundamental and might have evolved early on, at least in vertebrates. In line with this, Lane [4] writes that from the perspective of the evolution of life, ‘primordial emotions are widespread in vertebrates’. Lane discusses the work of Bjorn Merker [13], who argues that consciousness in hydranencephalic children demonstrates that consciousness cannot be essentially a cortical phenomenon. Lane says that: ‘One remarkable suggestion that consciousness is more widespread than we like to credit is the survival and apparent consciousness of those few exceptional children who are born without cerebral cortices’ [4, p. 258]. These children seem to be aware of primordial emotions that allow immediate reactions. Pain hurts because it alarms the cognitive system in ways that mere predictions concerning statistical probabilities of likelihood cannot capture. Moreover, with otherwise proper cognitive function, but without the experience of pain, each developmental milestone becomes an ordeal [4, p. 250]. As Lane says, ‘Pain is not alone. Hunger, thirst, fear, lust, these are among what Derek Denton calls the “primordial emotions” which he describes as imperious sensations that commandeer the whole stream of consciousness, compelling intentions to act’ [4, p. 250]. Thus, primordial emotions are also the foundation for normal development, physical and cognitive.

We take these observations to be critical and integrate them into one central claim of the ALARM theory of consciousness [8]: basic conscious experience, i.e. basic arousal, has the core function of alarming the biological agent in order to trigger a survival programme. This is essential for the living being if at least one of the various homeostatic regulation systems (regulation of breath, body temperature, food, water, sleep, etc.) is registered to be out of the range of tolerance, e.g. if the temperature is suddenly much too high. Since this means that the survival of the body is in danger, an immediate survival reaction is needed. Although the survival reaction can in principle be triggered without consciousness, there are several crucial advantages for a biological agent that has developed a conscious experience of pain to be connected with a sudden registration of an intolerable condition for the body; e.g. if an agent has a pain experience connected with burning the skin of the hand, then it cares for the hand as long as it hurts by not putting weight on it or grasping objects. This supports the healing process. Thus, primordial emotions were the first kind of consciousness that evolved in the animal world; they depend on non-cortical areas, and their function is, exactly, to powerfully commandeer the whole bodily system in order to prevent major harm or incur a non-remediable cost. We will discuss more advantages in the systematic overview of the functions of consciousness in §5.

Let us now put together the most important evidence in support of basic conscious experiences, i.e. basic arousal, as separate from a more advanced form of everyday consciousness in humans, which we call general alertness. As a starting point, we want to establish as a criterion of adequacy that basic arousal can be realized without the necessary involvement of cortical areas. This is especially relevant for the discussion of consciousness in non-human animals because it enables us to integrate sources of evidence for evolutionarily old and primary forms of consciousness. As already mentioned, Merker [13] highlights that hydranencephalic children are born without cortex, but most plausibly have very basic affective experiences of comfort and distress. In addition, he describes studies of cats with a completely removed cortex: they are still able to move purposefully, orient themselves in their surroundings by vision and touch (as do rodents) and are capable of solving a visual discrimination task in a T-maze [14]. More recent evidence comes from the findings by Redinbaugh *et al.* [15]. They confirm that specific thalamic activations are necessary for a basic type of conscious awareness. In particular, deep brain stimulation of the central lateral thalamus in anaesthetized macaques had the effect of waking them up [15]. This stimulation functions as an on–off switch. Stopping the stimulus had the immediate consequence for the apes of falling back to sleep or into anaesthesia again. Especially important is that the state they were in with stimulation can best be described as a state of basic arousal, i.e. they reacted to pain stimuli and perceptual stimuli, but they were not alert enough to be able to do any more cognitive tasks that demand focused attention, which they could do in normal wakefulness. This suggests that we should accept a basic form of consciousness, namely *basic arousal as a sensitivity for pain, pleasure and other basic affective states without focused attention*, that is distinct from normal wakefulness involving focused attention, which we call *general alertness*. This is developed in more detail with further evidence by Newen & Montemayor [8].

We highlight that the neural basis of consciousness can be independent from cortical processes, but we deny the radical interpretation of Merker [13] that this is always the case. The neural correlates of consciousness (NCCs) can be based on processes in the upper brainstem and thalamus, but this seems to be only the case for basic arousal [8,16,17]. As we will argue later, more complex forms of consciousness are in need of cortical processing, while this may nevertheless be strongly modulated by thalamic processes [17,18]. One remark about basic arousal may be in place concerning the worry that survival reactions can also be triggered unconsciously, and thus the alarming function may not be the central function of basic arousal. We defend an evolutionary trajectory according to which unconsciously produced survival reactions become connected with a conscious experience of pain, thereby producing several new survival advantages. If a person touches a hot stovetop, then there is a very fast reaction of withdrawing the hand, which is already initiated before the person is aware of the intense pain, but the additional pain experience at the level of basic arousal has the evolutionary advantages of: (i) continuing to keep the hand away from the stove (since it continues to hurt); (ii) the agent has this continuing signal to care for the body in relation to this injury by preferring long-term behaviour that supports the healing process; (iii) the pain experience is a much more general indicator of body challenges for a large variety of cases and new situations in contrast to unconscious triggering processes that allow only limited associations due to the characteristics of unconscious implicit learning.^1^ We also highlight (iv) that basic arousal already enables one-shot learning, which is typically not enabled with unconscious implicit associative learning.

### (b) Three phenomena of consciousness

The ALARM theory [9] proposes that we need a novel theoretical framework to account for the evolutionary functions of consciousness. As already described, a central claim of the ALARM theory is that we need to first distinguish two levels of phenomenal consciousness, namely basic arousal and general alertness. Basic arousal may be realized independently from cortical processes and it functions as a specific alarm system, keeping a biological organism alive under sudden intense threats. General alertness is a type of consciousness which involves the ability to focus on one sensory signal out of several, e.g. if a biological agent has a visual and an auditory input at the same time, then the ability to attentively focus on one of the signals (and change this focus) is a paradigmatic case of general alertness that can also be characterized as attention-guided consciousness. It is, in fact, what we humans experience as our everyday consciousness, but it is also clearly proven to be realized in many mammals, e.g. in mice [20].

From the evolutionary and synchronic functional perspective, we can distinguish two roles of consciousness: one for basic arousal based on the evolutionarily older brain systems and one for general alertness based on additional evolutionarily younger cortical structures. The evolutionarily old functional role of basic arousal is to trigger an alarm signal in the biological system, which can then start immediate survival reaction programmes. The evolutionarily younger (synchronic) functional role of general alertness is to enable or accelerate specific learning processes by selecting contexts and associating features of situations, forming an understanding of new regularities (causal or social rules). We will discuss these functional roles in more detail (see §5). Furthermore, we want to add as a new challenge the characterization of a third type of conscious phenomenon that we need to integrate into the evolutionary discussion of functions of consciousness, namely *reflexive consciousness*. Before discussing the functions of the three phenomena of consciousness, we want to establish the following characterization: *reflexive consciousness is consciousness involving metacognition about oneself or another person as an agent, while the content of the metacognition is a cluster of the agent’s states and processes, especially its mental states involving emotions, desires, beliefs etc. Let us call this cluster of mental states and processes of a person the mindset of a person*.

## 3. Reflexive self-consciousness

### (a) Reflexive self-consciousness can also be tested non-linguistically

Reflexive self-consciousness is prototypically realized when a biological agent is aware of its properties and abilities. When someone thinks an ‘I’-thought, e.g. ‘I am aware that I want to go to Hamburg next weekend’, this is an instantiation of a complex form of reflexive self-consciousness involving an awareness of one’s own desire. Reflexive self-consciousness of a desire is different from just having a conscious desire. Only the former is sufficient to allow one to start inhibiting a strong desire or to compare or modify the weights of different desires (on the basis of internal cognition) to improve controlled action. Reflexive self-consciousness needs the awareness of one’s state as one’s own (de se representation; [21]), but this need not involve linguistic representations, e.g. mirror self-recognition is an example from early cognitive development [22]: children of age 18 months usually are able to pass the typical mirror self-recognition task as well as great apes, elephants, dolphins, magpies and other species, but the ability remains realized only in a few animal species [23]. Another task illustrating a non-linguistic test of reflexive self-consciousness is the metacognitive task in which someone has to evaluate their own ability to memorize a number of photos correctly [24]: chimpanzees were presented with one picture first, and then, after a variable delay period, they were shown four pictures that either included the original one or not. Monkeys had to select the recently seen image in this match to a sample paradigm among the set of distracter images. Correct answers led to a high food reward. Incorrect answers resulted in no reward. In the critical version of the game, the chimpanzees were offered an opt-out button: instead of evaluating the test photo, they could press the opt-out option and receive a small food reward for leaving behind both the chance of a high reward and that of losing everything. Interestingly, they used the opt-out button depending on the delay time: with short delays, they went for the memory test, while with a longer delay time (with a certain changing point), they preferred to press the opt-out button, indicating that they had meta-knowledge that they had not memorized the earlier photo reliably enough. Furthermore, the test arrangements aimed to rule out ‘cueing’ by a wide variety of environmental and behavioural stimuli. Thus, the most plausible interpretation of the study is that the monkeys have the ability to selectively decline memory tests when they have forgotten the stimulus, and that they have basic awareness of their own memory ability [25].^2^ Given these two non-linguistic tests of *reflexive self-consciousness*, we need to account for this phenomenon in the context of evolutionarily early forms of consciousness realized independently from linguistic abilities, observable in non-human animals. How is it related to basic arousal and general alertness?

### (b) Reflexive self-consciousness is a case of general alertness with the activation of self-relational content involving metacognition

In the following, we argue that reflexive self-consciousness is not a third independent form of phenomenal consciousness, but a specific form of general alertness, namely one in which the content of the typical selective and goal-directed attention is information not about the world but about the biological agent itself, and this self-related content involves metacognition. Thus, reflexive self-consciousness is general alertness with self-related contents involving metacognition. We defend this claim with the following supporting arguments:

(i) (**ia**) The close intertwinement of self- and other-related contents: if a biological agent acts in the world and a *sense of agency, ownership or perspectivity* is involved, then there is activated and processed information not only *about the world* but also *about the biological agent itself* (self-relational content). (**ib**) Each ability involving self-relational contents can unfold in basic and complex forms (if the biological agent has, in principle, the cognitive architecture that is necessary to unfold it).
(ii) If self-relational contents are realized in connection with basic arousal, then we have a pre-reflexive form of self-consciousness,^3^ and if self-relational contents are realized with general alertness and involve metacognition (or meta-representation), then this results in typical cases of reflexive self-consciousness.

It follows that the features enabling reflexive self-consciousness are general alertness in combination with metacognitive processing of self-relational contents. Thus, although it is a third phenomenon, with a new functional role—as we will see—it does not involve a new type of conscious experience: we do not need to presuppose a new phenomenal quality. Attention-guided consciousness seems at least phenomenologically to be the only form of consciousness going beyond basic arousal that we as humans have. Then, self-consciousness relies on general alertness. The specific modification making self-consciousness a unique phenomenon consists only in the representational format, namely metacognition, and the type of content, namely self-relational contents.

About (**ia**): Self-relational contents are typically activated in cases in which a cognitive system has a sense of agency or a sense of ownership (of body parts or one’s mental states) or a sense of perspectivity (sensory, visual or cognitive perspective taking) [28]. In all cases of these cognitive abilities, we receive information both about ourselves and also about the world. From birth onwards, at the latest when a baby learns to grasp an object (within three months), while grasping it, the baby gains information about the object but also about its own hand; this is part of acquiring a basic form of self-representation of one’s body, enabling a self-world distinction (details in [29]). This is especially transparent when babies are lying on their backs, grasping their own feet. This is part of improving their body schema by developing a sensorimotor representation of their own body parts. Babies unfold their *sense of ownership* of body parts and learn which parts belong to the body and which objects are part of the world when they grasp themselves in contrast to a puppet. During this process of grasping the puppet, the sensory neural signals reaching the brain do not separate the information about the baby’s own hand and about the puppet. The brain has to do this job and probably does it with prior expectations about the bodily self and the puppet [29]. During this process, neural representations have to be projected either onto one’s own body or onto an object in the world [30]. This projection can lead to systematic failure, e.g. in the rubber hand illusion when somatosensory activity is projected not on the real hand, but on the fake hand by unusual visuo-tactile synchrony [31]. The sense of ownership can develop from bodily ownership into a sense of ownership (sometimes called authorship) of one’s own thoughts [29]. In a similar way, there are basic and complex forms of *a sense of agency*. One of us argued elsewhere [29] that a basic self-world distinction can be made on the grounds of systematic contingencies between motor commands and tendon receptor activation. Whenever an organism actively moves, this efference–afference circle occurs. Hence, changes in the perceptual flow can be divided into two classes: those accompanied by this circle (self-caused changes) and those that are not (world-caused changes). In this way, a basic self-world distinction can evolve. This is the basis for both a common coding of action and perception and the unfolding of a sense of agency (also in line with the work on core cognition [32]), which from the beginning separates the registration of one’s own action from the registration of objects and the situation involved in the action. This most basic form of agency is, according to Tomasello [33], goal-directed agency.

About (**ib**): *Goal-directed agency* can develop into *intentional agency*, which involves intentions as plans for more complex goals and which can involve actions with several steps. Even more demanding is *rational agency*, which involves explicit decisions to realize one out of many actions [33]. The most demanding form of agency may be one in which a biological agent acts based on metacognitive awareness about the social and the self-guiding rules controlling the actions of an agent (*normative agency* according to Tomasello [33]). Finally, a similar parallel self–other differentiation unfolds concerning *perspectivity*, resulting in parallel activation of self-representations and worldly representations.^4^ In a simplified view, we can distinguish three important stages in the ontogeny of perspectivity, namely the joint attention stage after the nine-month revolution, a pretence and rule-based stage roughly starting between 18 months and 2 years of age, and the mindreading stage starting roughly with the 4-year revolution.^5^ During each stage, children acquire information about the world and about themselves. Nine-month-old babies acquire joint attention, which involves a representation of the shared visual perspective on an object with someone else. Thus, it includes a pre-reflexive self-representation of one’s own visual perspective by registering a triadic relation between two agents, both focusing on the same object. Such a pre-reflexive self-representation is enriched to explicit self-representations starting with 18 months of age when passing the mirror self-recognition test [22], as well as other visual perspective-taking tasks [37]. This basic form of explicit visual self-representation is then systematically enriched, leading finally to theory-of-mind understanding of others, which includes a self-understanding based on self-ascribing beliefs, in contrast to ascribing beliefs to others [38]. Findings show that the neural representations of self-beliefs, in contrast to ascribing beliefs to others, have not only some specific features but also overlapping neural processing areas [39,40].

This coarse-grained overview of ownership, agency and perspectivity is sufficient to illustrate two aspects of self-relational contents: first that the specific abilities connected with a self (sense of agency, ownership and perspectivity) are realized such that they typically go together with a co-activation of dual contents, namely of the self and of the world, and second, that there is in all these different dimensions of the self a development from basic forms to complex forms of self-relational contents.

Now, we want to argue for thesis (ii), which we repeat: *If self-relational contents are realized in connection with basic arousal, then we have a pre-reflexive form of self-consciousness; and if self-relational contents are realized with general alertness and involve metacognition (or meta-representation), then this results in typical cases of reflexive self-consciousness*.

We have shown that a sense of ownership of agency and perspectivity often goes together with some involvement of consciousness since some activations of self-relational contents are processed consciously. Now we want to be more precise in describing the role of basic arousal and general alertness. We focus on selected studies of versions of mirror self-recognition and perspectivity since there is substantial research that allows us to distinguish different cognitive phenomena depending on the involvement of basic arousal or general alertness. Concerning self-sensitivity or self-recognition in live video or mirrors, there is clear evidence for rather early self–other differentiation involving only basic arousal: in a study, four- and nine-month-old babies were placed facing a live image either of themselves or of another person. It was shown that from four months of age, infants appeared to perceive and act differently when facing the image of themselves or the image of mimicking others [41]. Thus, there is an early sensitivity for oneself in the live image, which is not yet connected to further controlled and goal-directed and intentional activities as we observe it in mirror self-recognition with 18-month-old children: they start to act in a goal-directed fashion not only in relation to the other (make them do something) but also in relation to themselves: they actually remove a Post-it from their own head as soon as they see it in the mirror, they enjoy observing themselves acting in front of the mirror, and they even develop feelings of embarrassment [22]. The latter indicates the involvement of *general alertness* due to the involvement of selective and goal-directed attention. Thus, mirror self-recognition is an *early form of reflexive self-consciousness*, while the early self-sensitivity of four- to nine-month-old babies only involves *basic arousal*.

We think of joint attention as an intermediate case: it involves general alertness in the sensitivity for the other agent, the object and the joint perspective, but only basic arousal concerning the own visual perspective. To not over-intellectualize the ability of joint attention, we evaluate it as involving two sensitivities, namely, on the one hand, *general alertness for a joint perspective*, which is connected with the infant’s ability to initiate joint attention, or of producing joint attention to realize a goal-directed action, e.g. making the parent do something (e.g. handing over an object). On the other hand, there seems to be only *a basic sensitivity in the form of basic arousal for one’s own perspective involved in joint attention*. The reason is that the attention-guided version of conscious sensitivity of perspectivity that demands an awareness of one’s own perspective as being different from the others’ (visual perspective taking 1 [42]) is not yet developed and seems to unfold not earlier than 14 months of age [43].

There is further development of processing self-relational contents in ontogeny, as we described in the unfolding of the sense of perspectivity. Understanding one’s own *cognitive* perspective in contrast to others is fully developed only when the so-called explicit false belief task is passed at 4 years of age [35,44,45]. Then the self-relational contents involve beliefs, desires and other propositional attitudes in contrast to those connected to another person. On this basis, children develop a rich mindset of themselves (self-model) in contrast to person models of others [46], and this enables them to act in line with long-term self-directed plans and to realize and shape a self-model by acting in line with consciously reflected beliefs and desires. Thus, there is a new ability to learn, decide and act in relation to conscious meta-representations of oneself in present and future situations.^6^ Self-relational attitudes are just the prototypical case of reflexive self-consciousness we started out with, namely the conscious awareness of one’s own thoughts. This is analysed as a case of general alertness that is connected with complex self-relational contents involving meta-representations. It is paradigmatically realized with the ability of self-ascribing propositional attitudes.

We can summarize our observations as follows: a biological agent can unfold self-relational contents from rather simple to quite complex contents, while the latter are especially salient when the agent has self-relational beliefs, desires as well as further attitudes, which in the case of a linguistic agent may transform into a self-narrative. In normal human ontogenetic development, early forms of self-representations may remain unconscious (e.g. probably early forms of self–other distinctions) or only involve basic arousal (like in sensitivity for one’s own actions in a live video), while more complex forms of self-representations are typically connected with general alertness (like mirror self-recognition in humans and self-ascribing attitudes). As we have already noted, there are rather different behavioural abilities connected with each level of self-relational content being processed. The latter is essential for discussing the function of reflexive consciousness.

Let us pick up one of the central questions again: why do we not presuppose a third form of phenomenal consciousness (in addition to basic arousal and general alertness) for the phenomenon of reflexive self-consciousness, at least when combined with a complex self-relational content? This seems to be justified since it comes with new behavioural abilities for the biological agent, namely the agent can act in line with long-term self-directed plans to realize and shape a self-model by acting in accordance with consciously reflected mental states like beliefs and desires. Despite having a new functional role, we can more convincingly describe the phenomenon as relying on the already established form of general alertness that only receives specific contents, namely new self-relational contents, such as those involving meta-representations of oneself. It is mainly the ability of having and integrating meta-representations that boosts the function of general alertness, such that it enables new abilities like explicit *self-involving and future-directed* learning and decision-making. We do not introduce a third type of phenomenal consciousness for several reasons: (i) it is a more parsimonious explanation, (ii) there is neither phenomenological nor neural evidence that we need to introduce a third type of conscious experience going beyond general alertness, and (iii) it also fits with the neural data that conscious awareness of one’s own mindset overlaps substantially in neural activation with conscious awareness of others’ mindsets [39,40]. Since no-one considers introducing a new form of consciousness involved in awareness of others’ mindsets when the explicit false belief task is passed (but only a new type of content sensitivity), there are also no reasons to presuppose a new one involved in reflexive self-consciousness.

Let us summarize our arguments so far: although we need only distinguish two levels of phenomenal *conscious experience*, namely basic arousal and general alertness, we have introduced three functions of phenomenal consciousness. In addition to basic arousal and general alertness, we also include reflexive self-consciousness, each with a specific functional role. In the remaining sections, we first provide a short overview of the positions concerning the function of consciousness as discussed by IIT, HOT theories and GNWT. We demonstrate that none of them accounts for the early form of consciousness called basic arousal (§5). We then present a more detailed overview of the different and specific functional roles for each of the three phenomena of consciousness (§5), thereby providing a new basis for the investigation of various NCCs.

## 4. The underestimation of the plurality of functions of consciousness in select theories of consciousness

In the following, we focus on only two aspects of the three most dominant theories of consciousness, namely their predictions about the neural correlates and their commitments to the functional role of consciousness. From a birds-eye view, we want to highlight that despite all the differences, all three theories insist that neocortical areas are essential for processing phenomenal consciousness: IIT predicts a key role for processing conscious content in posterior cortex, whereas GNWT emphasizes a necessary role for the prefrontal cortex [47]. The predictions about neural correlates are less well determined for HOT theories. In the version of Lau [48], there are two neocortical regions involved, both representing the world and monitoring those representations, i.e. parietal and prefrontal cortices. As we have argued already, we think that the common claim of these three theories about the essential role of at least some neocortical processes is inadequate given the evidence about the presence of consciousness occurring independently form neocortical processing. We now discuss the three positions only with respect to their specific claims about the function of consciousness to situate our account in this context: in our terminology, all three only discuss the foundation of general alertness, and they have a gap about the evolutionarily older functional role of basic arousal.

### (a) The integrated information theory

IIT is a phenomenology-based, axiomatic theory, with the central claim that consciousness is identical with a certain level of integrated information [49]. If this is understood as an *a priori* identity claim, then to integrate information is not the *functional role* of consciousness, according to IIT, but rather it is what consciousness consists in. If the identity claim is understood *a posteriori*, then we are left with the key open questions: (i) Which level of integration is sufficient for consciousness? and (ii) Is the level of integration really completely independent of the realization basis? Furthermore, the methodology of IIT implies that although the theory is compatible with an evolutionary approach, evolution does not play any significant role in justifying the introspectively based axioms of IIT. Moreover, the main metric of IIT, the *Φ* measure, is not constrained by any consideration concerning evolutionary functions, including cost functions. What the IIT measure *Φ* detects is rather the degree of integration among units of information in a system. One challenge is *modularity of the mind*, which can be understood as a loss of integration in terms of cognitive architecture. Since modularity is present in many accounts of the human mind [50–52] and especially in evolutionary perspectives on non-human minds [53], which most plausibly enjoy conscious experiences (like most mammals), it would seem that IIT stands against such accounts of the evolution of consciousness. IIT proponents could argue that the integration measure would still be sufficient despite modularity. But it remains that IIT is not a theory that is constrained by evolutionary considerations. Positively, it is compatible with any theory of evolution, but negatively, it is lacking any connection to evolutionary functions (owing to its axiomatic grounding), especially to the central evolutionary functional role of consciousness to support survival in acute challenges of homeostatic equilibria like lack of oxygen, water or food, etc., which relies only on non-cortical regions. Thus, a defender of IIT may argue either that these non-cortical areas are still processing information to a sufficient degree of integration (which needs to be empirically supported) or that they are in need of widening their account to integrate all the relevant and recent observations.

### (b) Higher-order thought theories

The discussion of the function of consciousness should, according to Rosenthal [54], account for the distinction between creature consciousness and mental state consciousness. The former leads to the acceptance of a trivial function of being conscious for a biological agent as opposed to being asleep or unconscious, namely being able ‘to interact with its environment in ways that greatly enhance its well-being and survival’ [54, p. 832]. Concerning mental state consciousness, Rosenthal is much more sceptical and only admits an insignificant causal role: ‘Some states and processes may arise not because they afford reproductive or other advantages, but because they are by-products of psychological processes already in place. Given the difficulty of finding any credible function for the consciousness of psychological states, it is likely that such consciousness arises in that second way. The HOT theory suggests just such an explanation’ [54, p. 833]. In the most recent version of HOT, Lau [48] postulates a prefrontal perceptual reality monitoring (PRM) mechanism, which has the function to evaluate sensory areas as being either noise or driven by sensory stimulation. If PRM detects it as perceptual information, then a first-order state becomes phenomenally conscious. In his account, reality monitoring is the function of consciousness. The evaluation of this claim depends on the way reality monitoring is understood: if focused only on the result, namely the registration of data that an agent is disposed to act on, then such a registration process does not need conscious experience: evidence from blindsight, and from the dual processing of vision [7,55] demonstrates that unconscious registration of spatial information can be action-guiding. If it is understood in a more demanding sense, then it matches what we describe either as general alertness, or as a monitoring kind of reflexive consciousness. We leave this open and can accept an attention-guided awareness of reality for guiding actions as one function of general alertness. We would only need to add more central functions, especially upgraded learning abilities. Furthermore, we highlight a gap about an evolutionarily old functional role of basic arousal for HOT theories from both a neural processing and a functional perspective.

### (c) Global neuronal workspace theory

GNWT is essentially a functionalist theory that explains the function of consciousness as informational integration dependent upon neuronal signal-boosting; more precisely, its functional role is to make information realized in one part of the brain available to other parts of the brain, thereby making this information accessible to other cognitive subsystems. *Global* accessibility means availability to many other parts of the cognitive system, e.g. to integrate and broadcast information such that it enables higher cognitive functions like reasoning, decision-making and the formation of long-term memories. Given the focus on ‘global availability’, GNWT is a theory of *conscious access*, i.e. concerning contents that are in principle reportable, and if Block [56] is right, then this does not cover phenomenal consciousness completely. Now, given the focus on access consciousness, GNWT delivers a synchronic characterization of the function of access consciousness, which is what we describe as general alertness, but it ignores a phylogenetic perspective of more primitive forms of consciousness that we describe as basic arousal. Dehaene [57, ch. 3] discusses the function of access consciousness in more detail, with the core claim that consciousness is used to keep information as long as we need it, to compress information (e.g. into non-linguistic concepts or symbols) and to transfer compressed information to new and higher levels of processing. These advanced functional roles contribute to an explosion of learning abilities. This is in line with our analysis of the functional role of general alertness in the ALARM theory, but there is more to say about the primitive and more advanced phenomena of consciousness, namely basic arousal, on the one hand, and a third function, reflexive consciousness, on the other. This is a gap in GNWT.

## 5. The various functions of phenomenal consciousness: expanding the ALARM theory

Our central aim is now to provide a systematic and detailed overview of the various functions of three core phenomena of (phenomenal) consciousness, namely basic arousal, general alertness and reflexive consciousness (of the mindsets of oneself and others). We are convinced that this approach helps us make progress in investigating NCCs and their enabling functions in humans and non-human animals.

According to the ALARM theory [8], *basic arousal* functions as a specific alarm system, keeping a biological organism alive under sudden, intense threats. Let us unfold this: all homeostatic processes, like temperature regulation, are extremely relevant for survival, and they are the basis of the evolution of affective processing [58]. In normal, slow-changing environments, homeostatic regulation processes are realized in a kind of slow updating, e.g. if the temperature rises, then increased sweating is activated to keep the body temperature in the range of tolerance. But, when confronted with a radical challenge, e.g. suddenly the living being enters an area of very high temperature, slow updating no longer secures survival. This process needs to be stopped and a new one activated: this can be done by giving the incoming extreme temperature signal 100% weight and thereby triggering an immediate survival reaction, typically of flight. This has a big evolutionary advantage. In principle, such a mechanism can be implemented without consciousness: in the case of touching a hot stove with one’s hand, withdrawal of the hand sets in faster than the pain experience. But if the signal is registered with basic arousal as pain, then this makes a radical difference for the person: if a person feels pain, the agent not only withdraws the hand but also stops putting it anywhere near the hot stove again. If there were no pain involved, the question arises why the agent should not do it again (as in the rare case of humans without any pain sensitivity, who do not live long [59]). Furthermore, as mentioned, since the pain continues, the agent cares for the hand and lets it rest to prevent pain and thereby supports the healing process. In addition, the learning process connected with pain experiences is more general than without basic arousal: the person not only learns to avoid hot stoves, which can burn the skin, but also avoids anything that, even for the first time, causes pain (without any multi-case associative learning), because pain indicates a threat for survival and triggers an intense avoidance behaviour. *Thus, basic arousal has three specific functional roles, which we describe with the general function of alarming the bodily agent, namely (i) triggering a survival behaviour, (ii) caring for the body, and (iii) initiating a generalized learning*.

The next type of phenomenal consciousness is *general alertness*. The most important improvement is that it involves selective and goal-directed attention such that the biological agent can now select and focus much more on aspects of a situation, e.g. in a standard situation, a biological agent often has auditory and visual inputs, sometimes also further sensory inputs. General alertness allows, e.g. to focus on one aspect of the visual experienced situation. This enables the agent to learn much more specific regularities about the environment (e.g. indicators of food resources or further causal regularities). Thus, general alertness results in a boost of much flexible learning and decision-making, which enables an agent to develop new behavioural strategies in challenging situations. This boost of learning abilities presupposes that, through general alertness, the agent is enabled to keep a sensory input for some time and focus on it, then to compress the information to store the relevant aspects of the situation and, as Dehaene [57] highlights, to transfer compressed information to new and higher levels of processing, enabling the representation of new regularities. Thus, while basic arousal only needs to rely on short-term and procedural memory, general alertness is connected to the interaction of short-term memory with long-term memory. The interaction of selective and goal-directed attention with the memory systems enables an agent with general alertness to form advanced representations of the world, including the agent itself and other agents but not yet involving a conscious representation of the long-term mindset of an agent, i.e. there is not yet a conscious representation of background affective dispositions, beliefs, desires and other attitudes of the agent. Thus, general alertness enables the advanced representation of the world, of goal-directed intentions and actions of an agent expressed in a situation, but not yet the long-term background mindset of an agent. Therefore, *the function of general alertness is to enable advanced learning and decision-making strategies about the environment, thereby opening up new action possibilities given a challenge by improving social interaction in the light of goal-directed actions of other agents as well, e.g. to improve coordinated joint action*.

A third phenomenon is *reflexive self-consciousness*. We clarified that it is not realized with a new level of consciousness but a special case of general alertness with specific new contents (self-related information) enabled by meta-representations of the mindset of oneself (see above). Nevertheless, reflexive self-consciousness has a new function since now the advanced learning strategies are receiving an additional boost by meta-representational contents, enabling an advanced understanding of the world integrating the own mindset and that of other agents. Since reflexive consciousness of the mindset of oneself is closely intertwined with the ability to consciously attribute a mindset to other agents, we characterize the functional role of reflexive consciousness of the mindset of oneself or others as a unitary package. Concerning the memory system, reflexive consciousness is based on an ability for advanced mental time travel, such that *reflexive consciousness has the dominant functional role of enabling future-directed long-term planning accounting for one’s own mindset or that of others. This leads to a boost in social learning in complex social environments,* which, e.g. finds an expression in reputation management, which we can observe, e.g. in ape groups [60].

Finally, we can illustrate the functions of consciousness through a managerial analogy since this helps characterize the interactions of the three functions of consciousness. The first and most influential ‘conscious manager’ of basic arousal has the essential role of preventing major (or even existential) costs to the system. A standard survival behaviour is activated according to sensing situational threats relying on rather rigid behavioural programmes without choices and regardless of long-term planning and learning. The next more specialized manager, general alertness, controls the first-level signals of basic arousal in order to modulate them through top-down attention routines required for new habituations and long-term learning. In order to learn and habituate, general alertness needs input from areas concerning higher cognition, likely all cortical, integrating them with basic signals concerning homeostatic functions. Choices emerge from this integration. The two levels of influence are also described by Denton [61], who says that the ‘commandeering’ power of consciousness is twofold. In our terminology, the kind of cost avoidance involved in basic arousal does not involve a choice, while the next level of conscious ‘manager’, general alertness, depends fundamentally on choice in order to organize its cost–function operations, focusing on the environmental challenges: the plate I am holding is very hot, but if I drop it now, the cost could be worse than a small burn (e.g. I could get fired from my job or burn other people). When the signal is too strong, the second-level manager is overwhelmed by the urgency of basic arousal. But when conditions are normal, basic-level signals can be organized and attentively modulated in order to allow small immediate costs that pay off in the long run. The third-level manager is based on general alertness, but it includes meta-representations of oneself. Reflexive self-consciousness has additional long-term functions involving complex social abilities by integrating the mindset of oneself as well as that of others. This enables reputation management, advanced planning and, in linguistic beings, narrative self-models and person models. This is a presupposition of more stable and more highly predictable interactions in large social groups. In the case of holding a very hot plate, a person may still keep it in the hand, not because dropping it would have any negative consequences (like getting fired), but simply because the person wants others to appreciate how much pain she can stand. The three phenomena of consciousness and their functional roles are summarized in table 1.

**Table 1.** Three phenomena of consciousness and their functional roles

phenomenon of consciousness	phenomenality/ experience	specific attentional processes	dominant learning strategies	dominant memory resources	specific self-/ and other-involvement	dominant functional roles
reflexive consciousness about oneself or another person as having a mindset (involving mental states)	level-2 experience	self- and other-directed attention (including mindsets)	complex learning strategies involving meta-representations and the ranking of costs inlcudes the mindset of the self or others and future expectations	long-term and short-term memory with advanced mental time travel	meta-representation involving the mindset of oneself and other agents	long-term planning accounting for the mindset of oneself (self-model) or another person (person-model of others); boost of learning meta-features about oneself and others, e.g. in reputation management, narratives
general alertness	level-2 experience	selective and goal-directed attention	advanced learning strategies based on selective attention enabling new reactions (behavioural possibilities for challenging situations; complex cost functions concerning the ‘Umwelt’)	long-term and short-term memory	advanced representations about the world, oneself and others (but not yet about a mindset)	advanced learning and decision-making strategies about the environment, how to act in the environment and to socially interact
basic arousal	level-1 experience	signal-driven attention	simple associative learning (simple cost functions concerning the ‘Umwelt’)	short-term memory and procedural memory	simple representations about the world, minimal representations about oneself and other agents	alarming the cognitive system (the body/the agent): (i) triggering a survival behaviour; (ii) caring for the body; (iii) generalized learning
unconscious cognitive processing	no experience					survival in the environment

## 6. Conclusion

This layered view of consciousness helps us (i) to account for recent discoveries of subcortical brain activities with a central role of thalamic processes by integrating basic arousal, (ii) to distinguish the three phenomena of consciousness (all based on two levels of experience, namely basic arousal and general alertness), and (iii) to describe the three specific functional roles. We are able to describe the interaction of the three phenomena of consciousness and their functions such that phylogenetically later developed phenomena are based on earlier ones, and they can only operate if they are not being overridden by the older and more basic functions. Thus, there is not one function of phenomenal consciousness but at least three different functional roles (with several subfunctions) of three core phenomena of consciousness. *Basic arousal* has the core function of alarming the bodily agent, which includes (i) triggering a survival behaviour, (ii) caring for the body, and (iii) initiating a generalized learning. The function of *general alertness* is to enable advanced learning and decision-making strategies about the environment, thereby opening up new action possibilities given a challenge. The third phenomenon of consciousness, *reflexive self-consciousness* (or consciousness of other minds), enables future-directed long-term planning, accounting for one’s own mindset or that of others. Distinguishing three phenomena of phenomenal consciousness and their functional roles is an important basis to understand animal minds and can guide a more differentiated search for various distinct NCCs.

## Data Availability

This article has no additional data.

## Footnotes

^1^
The last aspect is closely connected with the idea of Ginsburg & Jablonka [19] that a basic form of consciousness that we call basic arousal is connected with unlimited associative learning (UAL). While we think that these increased learning abilities are a result of the evolution of basic arousal owing to a modified neural processing, Ginsburg & Jablonka argue that consciousness is constituted by UAL. For the purpose of the discussion of the functional role of basic arousal, we can ignore this difference. We agree that one functional role of basic arousal is a new quality of learning, which may be described as UAL, but we add more functional roles.

^2^
Despite some discussion, we think that opt-out studies can indicate metacognition [26]. The varieties of metacognitive evaluations and their relations to each other are clarified in [27].

^3^
A self–other differentiation is involved in the conscious experience, but the self not explicitly represented, e.g. the self is not consciously identified with an image as in mirror self-recognition.

^4^
We are not presupposing any problematic notion of a self as a substance or stable entity. Our background is the pattern theory of self that accounts for both, quite a variability as well as systematic changes of the self [34].

^5^
There are fine-grained differences in describing the main stages in cognitive ontology, but there seems to be also some main consensus that there are central turning points in cognitive development, which are sometimes described as revolutions with 9 months, 18–24 months and with 4 years of age [35,36].

^6^
Since humans are social beings, we do this always in the context of social interactions and social expectations.
